# Missouri State Dental Association

**Published:** 1891-12

**Authors:** Wm. Conrad


### PROCEEDINGS.

### Missouri State Dental Association.

The twenty-seventh annual meeting of the Missouri State Dental Association was held at Louisiana, Mo., July 7th to 10th inclusive.

The following officers were elected for the year 1892.

President, Dr. Geo. L. Shepard, Sedalia, Mo.; Vice-President, Dr. E. E. Shattuck, Kansas City, Mo.; Second Vice-President, Dr. J. T. Fry, Moberly, Mo.; Corresponding Secretary, Dr. Wm. Conrad, St. Louis, Mo.; Recording Secretary, Dr. W. M. Carter, Sedalia, Mo.; Treasurer, Dr. J. A. Price, Weston, Mo.

Board of Censors, Drs. C. J. McBride, Perryville, W. H. Buckley, Liberty, D. C. Lindsley, St. Louis, Mo.

Committee on Ethics, Drs. F. Slater, Rich Hill, E. B. Crane, California, E. W. Bear, Sedalia, Mo.

Publication Committee, Drs. E. E. Shattuck, Kansas City, W. S. Lowry, Kansas City, W. E. Tucker, Springfield, Mo.

Committee on Law, Dr. J. A. Price, Weston, Mo.

Committee on New Appliances, Dr. J. B. Vernon, St. Louis, Missouri.

The next meeting will be held at Clinton, Mo., the first Tuesday after July 4th, 1892.

WM. CONRAD, Cor. Sec'y.

